# Editorial: Organizational democracy, organizational participation, and employee ownership: Individual, organizational and societal outcomes

**DOI:** 10.3389/fpsyg.2023.1135138

**Published:** 2023-02-10

**Authors:** Wolfgang G. Weber, Christine Unterrainer, Thomas Faurhold Jønsson

**Affiliations:** ^1^Applied Psychology – Work and Organizational Psychology, Institute of Psychology, University of Innsbruck, Innsbruck, Austria; ^2^Department of Psychology and Behavioural Sciences, School of Business and Social Sciences, Aarhus University, Aarhus, Denmark

**Keywords:** organizational democracy, workplace democracy, participation, employee ownership, organizational behavior, collective decision-making, social sustainability, civic engagement

Organizational democracy (OD) refers to broad-based, and institutionalized employee participation that is not occasional in nature. Written rules, regulations and boards enable employees to exert influence on tactical and strategic decisions. This is realized through direct or representative co-determination or collective self-determination of the employees (e.g., worker cooperatives, self-governed firms). Additionally, employees often hold a share of their organization's equity capital (employee ownership) (Wegge et al., 2010; Weber et al., 2020). In contrast to research on decision making on the level of the individual job (e.g., job control) or the leadership dyad (e.g., participative goal setting) studies on the effects of democratic decision making or participation on higher levels exist only to a much lesser extent. Hence, this Research Topic aimed at collecting theoretical contributions, systematic research reviews and quantitative or qualitative empirical studies that help to clarify how OD, higher level participation or employee ownership are associated with psychological, organizational, or societal outcomes. Researchers have postulated that organizational participation and democracy would form a socialization field for personality development and societal responsibility through allowing employees to gather experiences in planning and decision making as well as mutual responsibility-taking (e.g., Pateman, 1970; Greenberg et al., 1996; Cohen, A., and Vigoda, 1998). Such a potential spillover effect is relevant for sustainable and democratic societies since we live in times of increasing debates about corporate corruption, the global environmental crisis, global inequality, or endangered societal cohesiveness.

Two articles in this Research Topic explicitly referred to this possible spillover effect. Rybnikova conceptually reflected Pateman (1970)'s spillover hypothesis and pointed to controversial considerations as empirical studies provided inconsistent evidence so far. The author identified main conceptual shortcomings for Pateman (1970)'s spill over hypothesis and provided avenues for future theoretical undertakings in analyzing whether and how employees' OD on the strategic level (in general meetings or representative boards) and their participation in lower organizational units (e.g., working groups) is related to participation in social and political domains. Such avenues encompass, e.g., employees' psychological ownership and moral development (Hannah et al., 2011), or the theoretical perspective of neo-institutionalism. The latter exemplifies how implicit cultural norms in a society may frame the status of workplace democracy as legitimate or not, fostering or inhibiting spillover effects as a result. Though, Breitling and Scholl showed in their empirical contribution that the one-sided economistic motivation concept of parts of neo-institutionalism is not able to explain prosocial organizational behavior of works councils.

Schümann et al. argued with the help of the relational job design framework (Grant, 2007) how and why the effects of a participative work climate spill over to employees' prosocial behavior outside the work context. In concrete terms, this means that employees take the working conditions of workers in the supply chain into account when selecting and buying goods. Analyses of two-wave data that were collected *via* an online panel questionnaire from 492 employees working in various industries in Germany provided evidence that the relationship between employees' individually perceived socio-moral climate and their socially responsible purchase intention was mediated by their perceived social impact. With their question of the extent to which a participatory organizational climate through socialization of employees can contribute in the long term to eliminating the global grievances of the unjust distribution of working and living conditions, the authors are treading on a still little cultivated scientific land. We would be pleased if their exploratory study could stimulate future research that also incorporates, through a multi-level design, the potential influence of strategic codetermination and employee ownership on global solidarity behavior.

Three contributions of this Research Topic focused on democratic enterprises, whose financial viability in a capitalist market environment has long been contested (e.g., “degeneration thesis” by Webb and Webb (1914); “iron law of oligarchy” by Michels, 1915). In a systematic review of 77 qualitative studies from nearly 50 years, Unterrainer et al. were able to disprove those deterministic hypotheses by showing that democratic enterprises can economically survive, prosper, and resist degenerative tendencies in the long run. For practitioners, organizational and external conditions, practices and psychological phenomena that contribute to the degeneration, regeneration, or resistance to degeneration were extensively described.

In a mixed-methods study on a large cooperative of the Spanish Mondragon Corporation Arregi et al. found that due to COVID-19 participation in decision-making has declined in certain governance channels. This was especially true for blue collar employees. On the other hand, degenerative tendencies could be countered, e. g., the General Assembly was implemented online after holding informative talks in small groups. COVID-19 had strengthened employees' commitment with the economic situation of the cooperative and acts of solidarity with colleagues indicating a sign of robustness and regeneration, since socially-oriented targets prevailed over profit ones.

Organizational commitment was also the focus of a qualitative study by Rodríguez-Oramas et al., centered on two big cooperatives of the Mondragon Corporation. The authors found three ways how democratic participation of worker-members may have contributed to the development of affective commitment: identification with the representatives of the governing bodies, intensive learning processes, and acknowledgment as co-owners and as part of collective business efforts. These antecedents correspond with factors supporting retention or regeneration of democratic enterprise structure that were identified in the systematic review by Unterrainer et al. like intensive cooperative education and training, open criticism and discussion and permanent requirement for accountability of managers, and, further, workers' strong commitment to cooperative idea.

The German model of co-determination through union representatives represents a further domain of this Research Topic. Breitling and Scholl investigated how works council and employee participation affected 45 organizational and process innovations in large businesses representing a wide spectrum of economic sectors. Qualitative case analyses revealed different profiles of works council participation depending on the innovation type. Quantitative analyses showed that both forms of participation delivered positive contributions to innovation success *via* knowledge growth. Furthermore, coordination capability partially mediated the relation only between works councils and innovation success. These results correspond with earlier findings on the positive impact on productivity that works councils supporting participation enhancing interventions demonstrated in a study representative for the German economy (Zwick, 2004).

Using a free association test, a survey and a vignette method, Wu et al. provided an intercultural comparison of preferences and perceptions of voice- and group-based workplace participation. Chinese and American employees differed in their construal of workplace participation, yet both groups valued the concept of participation positively. In both cultures participation was positively associated with the experience of productivity and job satisfaction, and negatively with workplace conflict. Finally, American employees favored a high-participation workplace and predicted positive outcomes, while Chinese employees were slightly more supportive of a low-participation workplace associating it with higher productivity.

The last three articles of this Research Topic deal with empirical studies that investigated possible effects of OD. Geçkil presented a focused systematic review based on 37 studies in different private and public enterprises that applied the multi-dimensional Organizational Democracy Scale in Turkey. The main results suggested that perceived OD was positively related with organizational citizenship behavior, organizational commitment, psychological capital, and job satisfaction, whereas OD was negatively related with job stress and organizational depression. This paper represents the first focused overview of quantitative relationships between experienced OD and organizational psychological outcomes in Turkey. Its results allow comparisons with findings on OD from other countries. On this basis, conclusions can be drawn about the expression of psychological phenomena under different political and cultural conditions.

The relationship between OD and meaningful work considering corporate social responsibility (CSR) as a potential mediator was investigated by Svendsen and Jønsson. The authors collected self-reported, cross-sectional, individual-level data (*N* = 204) at two measurement points from different nations and industries. The results of the SEM analyses confirmed that CSR perceptions partially mediated the relationship between OD and meaningful work. Hence, OD can play a direct role in increasing the experience of meaningful work, but also an indirect role trough employees' experience of CSR.

The final article of this Research Topic by Pap et al. deals with the protective function of a participative climate and supervisor support on service employees of a large clothing shop chain in Belgium. The authors applied a multi-level analysis and showed that participative climate (at the work-unit level) and supervisor support (at the employee level) significantly moderated the negative relationship between perceived customer incivility and job satisfaction.

Considering the articles gathered in this Research Topic together with existing research reviews from recent years (Lee and Edmondson, 2017; Battilana et al., 2018; Weber et al., 2020; Unterrainer et al.), at least the following research gaps and desiderata appear in addition to the new findings already mentioned:

(1) *The importance of participation and democracy in enterprises as a field of socialization for democracy in the society* has hardly been adequately researched empirically. Both conceptual (Rybnikova) and methodological problems have been clearly identified (Kim, 2021). Nevertheless, nearly no sophisticated longitudinal studies seem to exist of how socio-moral competencies and civic and political behavioral orientations can be fostered by democratic organizational structures and organizational practices, and what mediating psychological mechanisms and developmental processes play a role (cf. a meta-analysis by Weber et al. (2020)). Such longitudinal studies—with a contemporary theoretical-conceptual and methodological foundation—should also take into account the weight of other socialization instances in family, education and leisure time with regard to the outcomes mentioned.

(2) Even if the following tendency is not in the foreground of the contributions to this Research Topic, a *contrast to economistically reduced motivation theories* (which are based on postulates of the so-called rational choice paradigm and its axioms of hedonism, self-interest and egoistically directed social exchange) is noticeable in their majority. Authors of the present Research Topic refer in this respect to humanistically oriented theories like Deci and Ryan's self-determination theory, Pierce et al.'s psychological ownership theory, Bandura's social-cognitive theory, Kohlberg's approach of moral education, Grant's relational job design framework, Freeman's participation theory, and various offshoots of Pateman's spillover hypothesis. It seems to us an important task of future theoretical contributions in the field of organizational democracy research to investigate to what extent the included components of these theories are compatible with each other. To the extent that they are, appropriate theoretical elements could be integrated into a more complex theoretical framework. This could help to explore the interplay of societal and organizational conditions of democracy in the enterprise, participatory practices (knowledge exchange, planning, discourse, decision-making), collective and individual basic needs, values, motives, attitudes, experiences, and competencies, and resulting personal, organizational, and societal outcomes.

(3) Finally, the contributions *of* Geçkil, Svendsen and Jønsson, and Pap et al. suggest that the extent of organizational democracy experienced by employees correlates positively with indicators of wellbeing and negatively with health-related indicators. Both a meta-analysis (Weber et al., 2020) and a systematic review (Unterrainer et al.) provided evidence that the impact potential of substantive organizational democracy (as opposed to non-binding workplace participation and voice) in terms of self-actualization, wellbeing (with the exception of job satisfaction), and *psycho-physical health of workers has hardly been explored* so far. Looking at recent research reviews from the field of Positive Psychology (for example, the contributions to the Research Topic edited by Van Zyl and Salanova (2022)), it is striking that even within the conceptualizations of “positive organizations” or of “positive participatory organizational interventions,” employee-owned democratic enterprises and their typical collective communication and decision-making practices are completely ignored. As Battilana et al. (2018) have stated in their thorough conceptual review: “Yet surprisingly—as we will discuss—most of the discourse on alternative ways of organizing does not substantially invoke notions of democracy” (p. 259). Though, organizational democracy can be interpreted as a specific form of employees' control over their work environment. An extensive body of research has provided evidence that control on the level of the individual or the group plays in the prevention of work strain and impaired health (e.g., Theorell, 2004). Therefore, we would like to encourage psychological researchers to investigate whether control of employees at the level of the company is also an important resource not only for the overall wellbeing of the individual employee. It may also be a resource for the optimal functioning of the company and society according to the basic republican values of integrity, freedom, equality and fraternity.

## Author contributions

WW and CU wrote the original draft of this editorial jointly. TJ reviewed and revised it. All authors contributed to the article and approved the submitted version.
